# Multi-level determinants of failure to receive timely and complete measles vaccinations in Southwest China: a mixed methods study

**DOI:** 10.1186/s40249-021-00885-6

**Published:** 2021-07-22

**Authors:** Xian-Yan Tang, Man Cheng, Alan Geater, Qiu-Yun Deng, Ge Zhong, Yue-Dong Lin, Ning Chen, Tao Lan, Long-Yan Jiang, Man-Tong Zhu, Qiao Li

**Affiliations:** 1 Department of Epidemiology and Biostatistics, School of Public Health, Guangxi Medical University, China. No. 22nd, Shuangyong Road, Nanning, Guangxi Zhuang Autonomous Region 530021 People’s Republic of China; 2 Epidemiology Unit, Faculty of Medicine, Prince of Songkla University, Hat Yai, Thailand; 3 Institute of Vaccination, Guangxi Centre for Disease Control and Prevention, Nanning, Guangxi Zhuang Autonomous Region China

**Keywords:** Measles, Vaccination, Timeliness, Health system barriers, Mixed methods

## Abstract

**Background:**

Measles outbreaks re-emerged in 2013–2014 in Guangxi Zhuang Autonomous Region of China, where measles immunisation coverage is high. The discrepancy between the vaccination coverage and outbreaks indicates that timeliness is crucial, yet there is limited knowledge on the health system barriers to timely vaccination. Using integrated evidence at the household, village clinic, and township hospital levels, this study aimed to identify the determinants of failure in receiving timely measles vaccinations among children in rural Guangxi.

**Methods:**

A multi-stage stratified cluster sampling survey with a nested qualitative study was conducted among children aged 18–54 months in Longan, Zhaoping, Wuxuan, and Longlin counties of Guangxi from June to August 2015. The status of timely vaccinations for the first dose of measles-containing vaccine (MCV1) and the second dose of measles-containing vaccine (MCV2) was verified via vaccination certificates. Data on household-level factors were collected using structured questionnaires, whereas data on village and township-level factors were obtained through in-depth interviews and focus group discussions. Determinants of untimely measles vaccinations were identified using multilevel logistic regression models.

**Results:**

A total of 1216 target children at the household level, 120 villages, and 20 township hospitals were sampled. Children were more likely to have untimely vaccination when their primary guardian had poor vaccination knowledge [MCV1, odds ratio (*OR*) = 1.72; MCV2, *OR* = 1.51], had weak confidence in vaccines (MCV1, *OR* = 1.28–4.58; MCV2, *OR* = 1.42–3.12), had few practices towards vaccination (MCV1, *OR* = 12.5; MCV2, *OR* = 3.70), or had low satisfaction with vaccination service (MCV1, *OR* = 2.04; MCV2, *OR* = 2.08). This trend was also observed in children whose village doctor was not involved in routine vaccination service (MCV1, *OR* = 1.85; MCV2, *OR* = 2.11) or whose township hospital did not provide vaccination notices (MCV1, *OR* = 1.64; MCV2, *OR* = 2.05), vaccination appointment services (MCV1, *OR* = 2.96; MCV2, *OR* = 2.74), sufficient and uniformly distributed sessions for routine vaccination (MCV1, *OR* = 1.28; MCV2, *OR* = 1.17; MCV1, *OR* = 2.08), or vaccination service on local market days (MCV1, *OR* = 2.48).

**Conclusions:**

Guardians with poor knowledge, weak beliefs, and little practice towards vaccination; non-involvement of village doctors in routine vaccinations; and inconvenient vaccination services in township hospitals may affect timely measles vaccinations among children in rural China.

**Graphical abstract:**

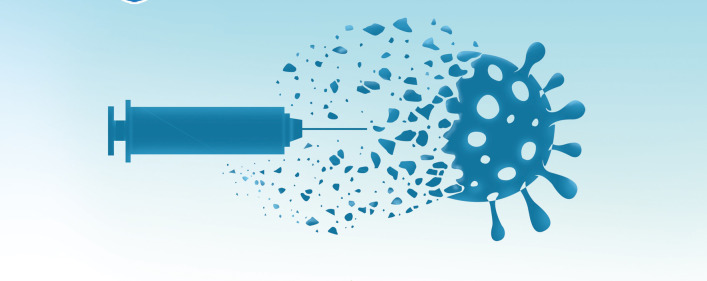

**Supplementary Information:**

The online version contains supplementary material available at 10.1186/s40249-021-00885-6.

## Background

Measles is a highly contagious disease that causes millions of pediatric deaths worldwide [1], however, it can be prevented with vaccines. Qualified vaccination with measles-containing vaccine (MCV) is a crucial and effective way to decrease the morbidity and mortality associated with measles infection among children [2]. Due to massive measles vaccinations worldwide since the 1980s, the MCV coverage has reached over 90% in most countries, particularly over 95% in developed countries including China, according to official reports [3]. Furthermore, the morbidity and mortality associated with measles have remarkably declined [4].

Nevertheless, while high vaccination coverage (≥ 95%) has been achieved in developed countries, the World Health Organization Europe Region (WHO/EUP) still reported large-scale outbreaks of measles in the United Kingdom, France, Spain, and Italy [5]. Most cases occurred in individuals with non-vaccination or incomplete vaccination, mainly aged ≤ 12 months or 15–29 years [6]. This implies that timely and complete measles vaccinations are critical in susceptible populations. Thus, global eradication of measles requires immunity in at least 95% of the susceptible population in each cohort, rather than a 95% mean coverage rate in the overall population. Likewise, frequent measles outbreaks in other WHO regions with high MCV coverage rates will delay measles elimination worldwide [7, 8].

China is a member of the WHO Western Pacific Region (WHO/WPR). Since 2003, China has accounted for approximately 70% of the reported measles cases in the WHO/WPR [9]. Therefore, the elimination of measles in China will largely contribute to measles elimination in the WHO/WPR. In recent years, large-scale outbreaks of measles have been reported in Guangxi, Zhejiang, Shandong, and Beijing despite ≥ 95% MCV coverage [10–13]. There has been an increasing incidence of infantile measles since 2004, and it is speculated that this trend reflects delayed vaccinations in infants and the rapid loss of passively acquired maternal antibodies.

Guangxi is located in Southwest China, which is in proximity to the Association of Southeast Asian Nations (ASEAN). From 2013 to 2014, mass outbreaks of measles resurged among children aged ≤ 2 years in rural Guangxi, where the coverage rate reached over 95% [14]. Specifically, 33 counties had a prevalence of > 2 per 100 000. Records show that 952 (71%) and 1774 (56%) local cases in 2013 and 2014, respectively, constituted children in rural areas; approximately 60% and 50% of cases in the consecutive years constituted children aged ≤ 24 months [15]. Among all cases, the MCV coverage was determined to be 63% and 51% in 2013 and 2014, respectively. Furthermore, the timely vaccination coverage for the first dose of MCV (MCV1) at 8 to 12 months of age was only 23% in 2013 and 15% in 2014.

Generally, a high (≥ 95%) MCV coverage should confer protection in children against measles [16, 17]. However, in practice, the timeliness of vaccination is not usually considered in official records. The reported coverage rate may conceal substantial delays in vaccination and neglect of appropriate vaccination schedules [18, 19]. The outbreaks have reflected a lack of herd immunity against measles among children in rural Guangxi, indicating that the complete vaccination might not be routinely delivered to susceptible populations on time. Therefore, the timeliness of vaccination should be highlighted, as it may be a key factor in eliminating measles.

Routine vaccination is a primary public health service in China. Vaccination campaigns are typically implemented with multilateral participants and logistics, including vaccination service demanders [20], service providers [21], a vaccination-related health system [22], the immunisation policy [23], and spatial accessibility [24]. Specifically, the determinants at household level are associated with the demander of vaccination services, including the knowledge, attitude, confidence, and practice of primary guardians regarding timely measles vaccination, as well as their satisfaction with vaccination services. Determinants at the village level mainly consist of spatial accessibility to township hospitals, allocation of vaccination-related health resources, participation of village doctors, and the perceptions of the immunisation policy. At the township level, determinants mainly include vaccination service provider-related factors, the vaccination-related health system, standard operating procedures of vaccination services, and perceptions of immunisation policy. These aspects may jointly determine the vaccination status of the child, whether there is timely vaccination, delayed vaccination, or non-vaccination.

To our knowledge, while most of the previous studies focused on some of these aspects, few studies have systematically explored vaccination-related health system barriers to timely vaccinations. Furthermore, there remains a paucity of system studies on the multilevel determinants of failure to receive timely and complete measles vaccination among children. Thus, for better understanding of the mechanism of failure in these cases, a mixed methods study comprising a cross-sectional survey and a qualitative study was conducted to identify multilevel determinants of untimely measles vaccinations among rural children in Guangxi, Southwest China, using integrated evidence from household, village clinic, and township hospital levels.

## Methods

### Study setting

The study was conducted in the rural areas of the Guangxi Zhuang Autonomous Region, which is one of five autonomous regions with a typical mountainous landscape in Southwest China (Additional file 1: Appendix S1 The location of Guangxi in China). In this region, there are more than 10 ethnic groups residing with good harmony for millennia, including the largest, the Zhuang ethnic group, and the Han, Yao, and Miao ethnicities. Administrative units consist of 14 prefectures and 111 counties, with a 237 600 km^2^ area and a population of 49.6 million residents as of 2019. Geographically, Guangxi is close to the ASEAN member states. In this study, rural areas were defined as counties or county-level cities in Guangxi.

### Study design

This study was conducted between June and August 2015. It employed a mixed methods research design that combined cross-sectional and qualitative studies.

A stratified three-stage cluster sampling approach was employed for the cross-sectional study. All rural counties were classified into four strata based on quartiles of measles incidence from 2011 to 2013. One county each was randomly selected from the first (lowest incidence) to the fourth stratum (highest incidence), namely, the Longan, Zhaoping, Wuxuan, and Longlin counties. In cluster sampling, five towns per county and six villages per town were randomly selected. Finally, 10 households per village were randomly selected. The inclusion criteria for target children were as follows: (1) resided in rural Guangxi for ≥ 3 months, (2) aged 18–54 months during the survey period, (3) had available child vaccination certificate, and (4) had a primary guardian who could be responsible for immunisation of children and verbally communicate. Children were excluded if they had any contraindications for vaccination or if they received any dose of MCV outside of their hometown. Some parts of the “Study Design” are consistent with those in a previous study [15].

For the qualitative study, convenience sampling was performed. The study population consisted of vaccination-related healthcare workers, including village doctors at village clinics and health professionals in township hospitals. The inclusion criterion for healthcare workers was engagement in measles vaccination services for at least two years. Healthcare workers who were unwilling to participate in the study were excluded. One village doctor per village clinic and three health professionals per township hospital (i.e. one leader and two vaccination professionals) were sampled; a total of 120 village doctors and 60 health professionals were included in this study.

### Data collection and data management

At the household level, vaccination records, dates, and status (i.e. delayed vaccination, timely vaccination, early vaccination, and non-vaccination) for the first dose of MCV (MCV1) and the second dose of MCV (MCV2) were obtained from children’s vaccination certificates. Furthermore, we recruited guardians to answer the questionnaire for children. Data on household socio-demographic and socio-economic factors and primary guardian's knowledge, attitude, confidence, practice on measles vaccination, and overall satisfaction with vaccination service were collected through face-to-face interviews using structured questionnaires (Additional file 1: Appendix S2 Structured questionnaire at household level).

At the village clinic level, the data regarding vaccination-related health resource allocation, participation of village doctors in vaccination services, and perceptions of the current vaccination policy were extracted from the tape-recorded in-depth interviews and semi-structured questionnaires (Additional file 1: Appendix S3 Semi-structured questionnaire at village level). At the township hospital level, data regarding vaccination-related health resource allocation, vaccination provider-related factors, standard operating procedures of vaccination services, performance of vaccination-related health systems, and perceptions of the current vaccination policy were extracted from in-depth interviews, focus group discussions, and semi-structured questionnaires (Additional file 1: Appendix S4 Semi-structured questionnaire at township level).

In addition, the geographical coordinates of the village and township hospitals were obtained via global positioning system. Vector maps of road networks and counties at the village scale, and digital elevation maps of Guangxi were acquired from the Guangxi Bureau of Surveying, Mapping, and Geoinformation. These data were utilised to calculate the travel time and distance to the township hospital.

A database with suitable range checks and validation was developed in EpiData version 3.1 (EpiData Association, Odense, Denmark) to conduct double entry for the quantitative data from the cross-sectional survey and the quantitative/semi-quantitative data of the qualitative study.

Quality controls were implemented in the whole process of data collection and data management. Pre-interview, a 2-day training course was arranged for interviewers. The course facilitated interviewer understanding the objective of survey, the meaning of each item, the procedure of interview, and interviewing skills. During interview, enough time and a comfortable environment were ensured to conduct interviews. Appropriate introduction was given to help guardians respond to each item objectively. Post-interview, the integrity and validity of the data were verified on each survey day. And coding of items was done in the field.

### Measurement of variables

Timely vaccination for MCV1 is designated when the child receives the first vaccine dose at 8 months of age (244–273 days). Meanwhile, timely vaccination for MCV2 is designated when the child receives the second vaccine dose between 18 and 24 months of age (548–730 days). If a child received a vaccine dose before or beyond the recommended schedule, or if a child had not received any dose of MCV at the time of the interview, the child was deemed to have untimely vaccination. In this study, untimely vaccination was the dependent variable in the multilevel logistic regression model.

The independent variables were obtained at three levels. At the household level, the independent variables were socio-economic status indicators, socio-demographic characteristics, primary guardian's knowledge, attitudes, beliefs, and practices (KABP) toward measles vaccination, and primary guardian's satisfaction with the vaccination service. At the village level, the independent variables were involvement of village doctors in routine vaccination services and travel time to township hospitals. At the township level, independent variables included the status of full-time vaccination service providers, status of vaccination professional allocation, vaccination services on local market days, arrangement of formal appointments with primary guardians on the next vaccination date, provision of monthly vaccination notice sheets for children, number of sessions for routine vaccination per month, and distribution of regular vaccination sessions in a month. The measurement of the independent variables is presented in Additional file 1: Appendix S5 Measurement of independent variables .

### Statistical analysis

Since a multi-stage stratified cluster sampling approach was applied in this study, the data not only indicate a hierarchical structure (i.e. household level, village clinic level, township hospital level) but also highly suggest similarity in the socio-economic status and pattern of routine vaccination service within each level. Considering the data structure and characteristics, the multilevel logistic regression model was employed to identify the determinants of failure to receive timely and complete measles vaccination using the “R2MLwiN” package in R software version 3.4.2 (R Project for Statistical Computing, Vienna, Austria).

For best fit, the variances of the null model were compared at different levels (Additional file 1: Appendix S6 Parameter estimation for null models at different levels). In the one-level model (i.e. household level), constant variance was significant, implying that the multilevel model should be fitted for the hierarchical structure of the data. In the two-level model (i.e. village-household level), variance at the village level was significant, indicating that the random effect of village-level factors existed. In the three-level model (i.e. township-village-household level), township-level variance was significant, and nearly 80% of the village-level variance was attributed to that at the township level. The variance partition coefficient was 80.1% and 79.7% for MCV1 and MCV2, respectively. Currently, no statistical approach has been established to combine the sampling weights of cluster surveys into multilevel models. Thus, three-level logistic regression models were developed to fit the three-level data in this study.

## Results

### Characteristics of study samples (Table 1)

**Table 1:** Descriptive statistics of determinants by study level

Variable	Sample (*n*)		Percentage (%)	
Household level (*n* = 1216)				
Child’s status				
NLBC	652		53.6	
LBC	564		46.4	
Ethnicity				
Han	330		27.1	
Zhuang	763		62.7	
Other	123		10.1	
Household registration status				
Registered	1137		93.5	
Unregistered	79		6.5	
Siblings				
Yes	857		70.5	
No	359		29.5	
Primary guardian’s age (years)				
≤ 45	686		56.4	
≥ 46	530		43.6	
Primary guardian’s education				
Primary school or below	597		49.1	
Junior high school	527		43.3	
Senior high school or above	92		7.6	
Primary guardian’s occupation				
Farmer	1165		95.8	
Non-farmer	51		4.2	
Annual per capita family income (CNY)				
< 4800	376		30.9	
4800–8000	477		39.2	
8000	363		29.9	
Measles vaccination status of child’s mother				
Vaccinated	355		29.2	
Non-vaccinated	861		70.8	
Annual average duration of time father is away from home (months)				
≤ 6	593		48.8	
≥ 7	623		51.2	
Annual average duration of time mother is away from home (months)				
≤ 6	726		59.7	
≥ 7	490		40.3	
Received a pre-vaccination physical examination from healthcare worker				
Yes	940		77.3	
No	276		22.7	
Received an explanation of vaccination side effects from healthcare worker				
Yes	873		71.8	
No	343		28.2	
Received post-vaccination advice from healthcare worker				
Yes	732		60.2	
No	484		39.8	
Primary guardian’s knowledge score on measles vaccination				
≤ 8 (poor)	526		43.3	
≥ 9 (good)	690		56.7	
Primary guardian’s perception of susceptibility to measles (score)				
≤ 18 (poor)	705		58.0	
≥ 19 (good)	511		42.0	
Primary guardian’s perception of severity in measles (score)				
≤ 14 (poor)	713		58.6	
≥ 15 (good)	503		41.4	
Primary guardian’s perception of benefit from measles vaccination (score)				
≤ 15 (poor)	429		35.3	
≥ 16 (good)	787		64.7	
Primary guardian’s perception of barriers to vaccination (score)				
≤ 22 (weak)	717		59.0	
≥ 23 (strong)	499		41.0	
Primary guardian’s perception of cues to action (score)				
≤ 11 (few)	887		72.9	
≥ 12 (sufficient)	329		27.1	
Primary guardian’s perception of self-efficacy (score)				
≤ 12 (poor)	701		57.6	
≥ 13 (good)	515		42.2	
Primary guardian’s practice towards measles vaccination (score)				
≤ 12 (few)	631		51.9	
≥ 13 (sufficient)	585		48.1	
Primary guardian’s satisfaction with vaccination service (score)				
≤ 42 (low)	651		53.5	
≥ 43 (high)	565		46.5	
Village level (*n* = 120)				
Village doctor’s involvement in routine measles vaccination				
Yes	42		35.0	
No	78		65.0	
Travel-time to township hospital (minutes)				
≤ 29	100		83.3	
≥ 30	20		16.7	
Township level (*n* = 20)				
Provision of a written appointment service				
Yes	7		35.0	
No	13		65.0	
Offering monthly vaccination notice sheet				
Yes	7		35.0	
No	13		65.0	
Number of monthly sessions for routine vaccination (days)				
≤ 4	8		40.0	
≥ 5	12		60.0	
Sessions uniformly distributed over a month				
Yes	10		50.0	
No	10		50.0	
Provision of vaccination service on local market day				
Yes	12		60.0	
No	8		40.0	
Full-time vaccination workgroup				
Established	4		20.0	
Unestablished	16		80.0	
Met the allocation standard for vaccination professionals				
Yes	6		30.0	
No	14		70.0	

*NLBC* non-left-behind child, *LBC* left-behind child

At the household level, 1216 eligible children were surveyed. Among the primary guardians of the study participants, 58% had poor perception of susceptibility to measles, 58.6% had poor knowledge of measles severity, 35.3% had poor awareness of the benefits from measles vaccination, 41% perceived strong barriers to vaccination, 72.9% had insufficient vaccination notices, 57.6% had poor perception of self-efficacy, 51.9% had few practices towards measles vaccination, and 53.5% had a low degree of satisfaction with vaccination services provided by township hospitals.

At the village level, a total of 120 villages were sampled, wherein 65% did not have a village doctor involved in routine measles vaccination services. At the township level, a total of 20 township hospitals were included, wherein 65% neither provided a formal or written appointment service nor offered a monthly vaccination notice sheet, 40% did not provide vaccination services on local market days, and 50% did not uniformly distribute the regular sessions in a month.

### Determinants of untimely vaccination obtained from univariate model (Table 2)

**Table 2:** Determinants of untimely vaccination for MCV1 and MCV2 obtained from univariate model

Variable	MCV1			MCV2		
	*OR*	95% *CI*	*P* value^*^	*OR*	95% *CI*	*P* value^*^
Household level						
Child’s status			0.031			0.849
NLBC	1.00			1.00		
LBC	1.29	1.02–1.62		1.03	0.76–1.39	
Ethnicity			0.003			0.074
Han	1.00			1.00		
Zhuang	1.19	0.91–1.56		1.39	1.00–1.94	
Other	2.07	1.36–3.15		1.08	0.64–1.84	
Household registration status			0.011			0.077
Registered	1.00			1.00		
Unregistered	1.81	1.15–2.86		1.66	0.95–2.91	
Siblings			0.102			0.163
Yes	1.00			1.00		
No	0.81	0.63–1.04		0.78	0.55–1.11	
Primary guardian’s age (years)			0.047			0.956
≤ 45	1.00			1.00		
≥ 46	1.26	1.01–1.59		0.99	0.73–1.34	
Primary guardian's education			0.008			0.005
Primary school or below	1.00			1.00		
Junior high school	0.59	0.38–0.92		0.59	0.43–0.82	
Senior high school or above	0.50	0.32–0.78		0.98	0.57–1.71	
Primary guardian’s occupation			0.585			0.439
Farmer	1.00			1.00		
Non-farmer	1.17	0.67–2.06		1.31	0.66–2.62	
Annual per capita family income (CNY)			0.295			0.704
< 4800	1.00			1.00		
4800–8000	1.13	0.85–1.48		1.05	0.74–1.50	
> 8000	0.90	0.67–1.21		0.89	0.61–1.33	
Measles vaccination status of child's mother			< 0.001			< 0.001
Vaccinated	1.00			1.00		
Non-vaccinated	2.54	1.93–3.34		1.98	1.36–2.88	
Annual average duration of time father is away from home (months)			0.309			0.592
≤ 6	1.00			1.00		
≥ 7	1.13	0.89–1.42		0.92	0.68–1.25	
Annual average duration of time mother is away from home (months)			0.443			0.246
≤ 6	1.00			1.00		
≥ 7	1.09	0.87–1.39		0.83	0.61–1.14	
Received a pre-vaccination physical examination from healthcare worker			0.003			0.197
Yes	1.00			1.00		
No	1.50	1.15–1.97		1.26	0.89–1.77	
Received an explanation of vaccination side effect from healthcare worker			< 0.001			0.147
Yes	1.00			1.00		
No	1.76	1.36–2.26		1.27	0.92–1.75	
Received post-vaccination advice from healthcare worker			< 0.001			0.007
Yes	1.00			1.00		
No	1.88	1.48–2.37		1.51	1.12–2.04	
Primary guardian’s knowledge score on measles vaccination			< 0.001			< 0.001
≤ 8 (poor)	1.00			1.00		
≥ 9 (good)	0.32	0.26–0.41		0.41	0.30–0.56	
Primary guardian’s perception of susceptibility to measles (score)			< 0.001			< 0.001
≤ 18 (poor)	1.00			1.00		
≥ 19 (good)	0.57	0.45–0.73		0.66	0.48–0.91	
Primary guardian’s perception of severity of measles (score)			0.308			0.663
≤ 14 (poor)	1.00			1.00		
≥ 15 (good)	0.89	0.70–1.12		0.93	0.69–1.27	
Primary guardian’s perception of severity of measles (score)			0.308			0.663
≤ 14 (poor)	1.00			1.00		
≥ 15 (good)	0.89	0.70–1.12		0.93	0.69–1.27	
Primary guardian’s perception of benefit from measles vaccination (score)			0.839			< 0.001
≤ 15 (poor)	1.00			1.00		
≥ 16 (good)	0.97	0.77–1.24		0.57	0.42–0.78	
Primary guardian’s perception of barriers to vaccination (score)			< 0.001			< 0.001
≤ 22 (weak)	1.00			1.00		
≥ 23 (strong)	5.82	4.53–7.49		3.49	2.55–4.79	
Primary guardian’s perception of cues to action (score)			< 0.001			< 0.001
≤ 11 (few)	1.00			1.00		
≥ 12 (sufficient)	0.41	0.31–0.54		0.25	0.15–0.40	
Primary guardian’s perception of self-efficacy (score)			0.016			0.111
≤ 12 (poor)	1.00			1.00		
≥ 13 (good)	0.75	0.59–0.95		0.78	0.57–1.06	
Primary guardian’s practice towards measles vaccination (score)			< 0.001			< 0.001
≤ 12 (few)	1.00			1.00		
≥ 13 (sufficient)	0.06	0.04–0.08		0.16	0.11–0.24	
Primary guardian’s satisfaction with vaccination service (score)			< 0.001			< 0.001
≤ 42 (low)	1.00			1.00		
≥ 43 (high)	0.32	0.25–0.40		0.52	0.38–0.72	
Village level						
Village doctor’s involvement in routine vaccination service			< 0.001			< 0.001
Yes	1.00			1.00		
No	1.64	1.28–2.10		2.05	1.45–2.91	
Travel-time to township hospital (minutes)			0.011			0.066
≤ 29	1.00			1.00		
≥ 30	1.89	1.16–3.09		1.81	0.96–3.39	
Township level						
Provision of a written appointment service			< 0.001			< 0.001
Yes	1.00			1.00		
No	2.39	1.67–3.42		3.23	1.79–5.81	
Offering monthly vaccination notice sheet			< 0.001			< 0.001
Yes	1.00			1.00		
No	1.64	1.28–2.10		2.05	1.45–2.91	
Number of monthly sessions for routine vaccination (days)			0.040			0.307
≤ 4	1.00			1.00		
≥ 5	0.78	0.62–0.98		0.85	0.63–1.16	
Sessions uniformly distributed over a month			0.007			0.904
Yes	1.00			1.00		
No	1.37	1.09–1.73		1.02	0.75–1.38	
Provision of vaccination service on local market day			< 0.001			0.436
Yes	1.00			1.00		
No	1.92	1.51–2.43		1.13	0.83–1.53	
Full-time vaccination workgroup			0.005			0.011
Established	1.00			1.00		
Unestablished	1.50	1.13–1.99		1.57	1.11–2.22	
Met the allocation standard for vaccination professional			0.715			0.012
Yes	1.00			1.00		
No	1.05	0.81–1.35		1.52	1.09–2.11	

*OR* odds ratio, *NLBC* non-left-behind child, *LBC* left-behind child

* *P* value in LR test

At the household level, children who were left behind in their rural hometown (MCV1, *OR* = 1.29), who belonged to a minority ethnicity (MCV1, Zhuang, *OR* = 1.19; MCV1, Other, *OR* = 2.07), who were unregistered in the household system (MCV1, *OR* = 1.81), or whose mother was non-vaccinated (MCV1, *OR* = 2.54; MCV2, *OR* = 1.98) were significantly less likely to receive timely vaccination. Children whose primary guardian was aged ≥ 46 years (MCV1, *OR* = 1.26), had a low level of educational attainment (MCV1, *OR* = 2.00; MCV2, *OR* = 1.02), had poor vaccination knowledge (MCV1, *OR* = 3.13; MCV2, *OR* = 2.44), had poor perception of susceptibility to measles (MCV1, *OR* = 1.75; MCV2, *OR* = 1.51), had poor awareness of benefits from measles vaccination (MCV2, *OR* = 1.75), perceived strong barriers to vaccination (MCV1, *OR* = 5.82; MCV2, *OR* = 3.49), had insufficient vaccination notices (MCV1, *OR* = 2.43; MCV2, *OR* = 4.00), had poor perception of self-efficacy (MCV1, *OR* = 1.33), had few practice towards measles vaccination (MCV1, *OR* = 16.66; MCV2, *OR* = 6.25), or had a low degree of satisfaction with vaccination service (MCV1, *OR* = 3.13; MCV2, *OR* = 1.92) similarly had significantly less likelihood of receiving timely vaccination.

At the village level, children living in villages where the travel time to township hospitals was long, defined as ≥ 30 min (MCV1, *OR* = 1.89) and whose village doctors did not take part in routine vaccination services (MCV1, *OR* = 1.64; MCV2, *OR* = 2.05) were significantly less likely to receive timely vaccination.

At the township level, children whose township hospital did not provide a formal appointment service (MCV1, *OR* = 2.39; MCV2, *OR* = 3.23), a monthly vaccination notice sheet, or vaccination services on local market days (MCV1, *OR* = 1.92) were less likely to be vaccinated in a timely manner. Lastly, children whose township hospitals had a few regular sessions for routine vaccination (MCV1, *OR* = 1.28), a clustered distribution of sessions within a month (MCV1, *OR* = 1.37), an unestablished full-time vaccination workgroup (MCV1, *OR* = 1.50; MCV2, *OR* = 1.57), or did not meet the allocation standard for vaccination professionals (MCV2, *OR* = 1.52) were significantly less likely to receive timely measles vaccination.

### Determinants of untimely vaccination obtained from multilevel model (Table 3)

**Table 3:** Determinants of untimely vaccination for MCV1 and MCV2 obtained from multilevel model

Variable	MCV1		MCV2	
	*OR* (95% *CI*)	P value*	*OR* (95% *CI*)	*P* value*
Household level (level-1)				
Primary guardian’s knowledge score on measles vaccination		< 0.001		0.031
≤ 8 (poor)	1.00		1.00	
≥ 9 (good)	0.58 (0.44–0.79)		0.66 (0.45–0.96)	
Primary guardian’s perception of susceptibility to measles (score)		0.038		0.048
≤ 18 (poor)	1.00		1.00	
≥ 19 (good)	0.76 (0.57–0.98)		0.70 (0.50–0.99)	
Primary guardian’s perception of benefit from measles vaccination (score)				0.020
≤ 15 (poor)			1.00	
≥ 16 (good)			0.65 (0.46–0.94)	
Primary guardian’s perception of barriers to vaccination (score)		< 0.001		< 0.001
≤ 22 (weak)	1.00		1.00	
≥ 23 (strong)	4.58 (3.44–6.09)		2.66 (1.83–3.84)	
Primary guardian’s perception of cues to action (score)		0.081		< 0.001
≤ 11 (few)	1.00		1.00	
≥ 12 (sufficient)	0.74 (0.52–1.03)		0.32 (0.18–0.55)	
Primary guardian’s perception of self–efficacy (score)		0.156		
≤ 12 (poor)	1.00			
≥ 13 (good)	0.78 (0.56–1.09)			
Primary guardian’s satisfaction with vaccination service (score)		< 0.001		< 0.001
≤ 42 (low)	1.00		1.00	
≥ 43 (high)	0.49 (0.37–0.68)		0.48 (0.34–0.69)	
Primary guardian’s practice towards measles vaccination (score)		< 0.001		< 0.001
≤ 12 (few)	1.00		1.00	
≥ 13 (sufficient)	0.08 (0.05–0.12)		0.27 (0.16–0.43)	
Village level (level-2)				
Village doctor’s involvement in routine vaccination service		0.013		0.025
Yes	1.00		1.00	
No	1.85 (1.14–2.99)		2.11 (1.09–4.04)	
Travel-time to township hospital (minutes)		0.125		0.226
≤ 29	1.00		1.00	
≥ 30	1.62 (0.87–3.02)		1.38 (0.82–2.32)	
Township level (level-3)				
Provision of a written appointment service		< 0.001		0.033
Yes	1.00		1.00	
No	2.96 (1.81–4.84)		2.74 (1.08–4.55)	
Provision of vaccination service on local market day		< 0.001		
Yes	1.00			
No	2.48 (1.52–4.08)			
Sessions uniformly distributed over a month		0.004		
Yes	1.00			
No	2.08 (1.25–3.45)			
Full-time vaccination workgroup		0.453		0.298
Established	1.00		1.00	
Unestablished	1.16 (0.78–1.73)		1.49 (0.70–3.19)	
Met the allocation standard for vaccination professional				0.170
Yes			1.00	
No			1.43 (0.75–2.75)	
Variance components				
Township level variance	0.048^#^	0.201^#^		
Village level variance	0.091^#^	0.087^#^		
Household level scale parameter	1.000	1.000		

* Penalized Quasi-likelihood (PQL) test

^#^*P* > 0.05

**Fig. 1:**
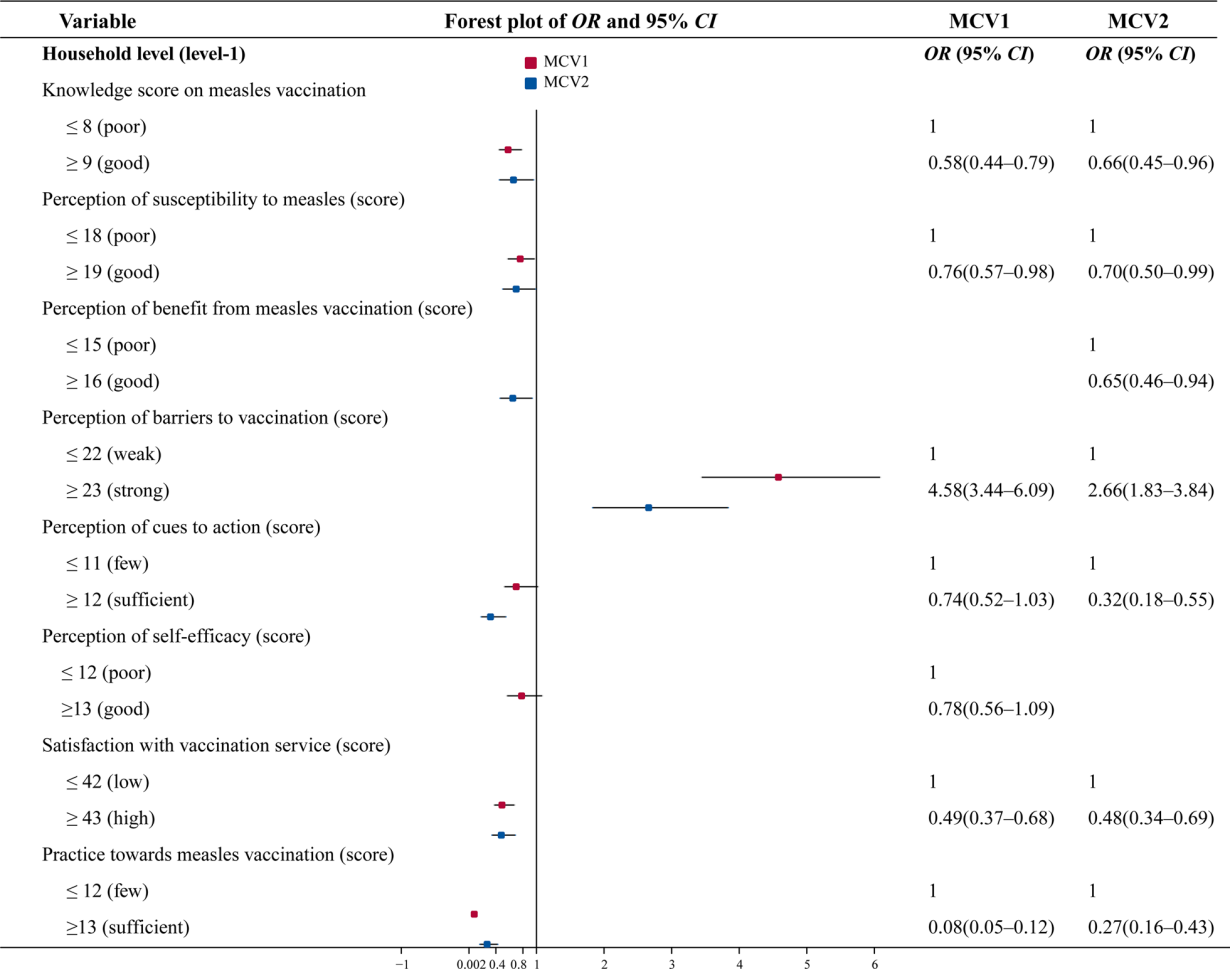
Forest plot of multilevel logistic regression, for the determinants of untimely vaccination of the first dose of measles-containing vaccine (MCV1) and the second dose of MCV (MCV2) at household level. *OR* odds ratio, *CI* confidence interval

At the household level, children whose primary guardians had poor vaccination knowledge (MCV1, *OR* = 1.72; MCV2, *OR* = 1.51), had poor perception of susceptibility to measles (MCV1, *OR* = 1.31; MCV2, *OR* = 1.42), had poor awareness of benefits from measles vaccination (MCV2, *OR* = 1.53), perceived strong barriers to vaccination (MCV1, *OR* = 4.58; MCV2, *OR* = 2.66), had insufficient vaccination notices (MCV2, *OR* = 3.13), had few positive practices toward measles vaccination (MCV1, *OR* = 12.5; MCV2, *OR* = 3.70), and had a low degree of satisfaction with vaccination services (MCV1, *OR* = 2.04; MCV2, *OR* = 2.08) were significantly less likely to receive timely vaccination (Fig. 1).

**Fig. 2:**
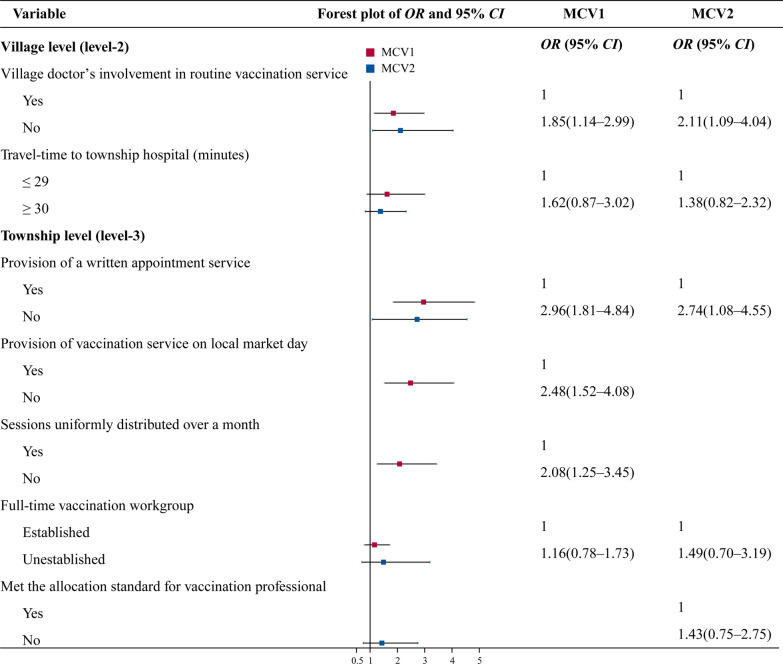
Forest plot of multilevel logistic regression, for the determinants of untimely vaccination of the first dose of measles-containing vaccine (MCV1) and the second dose of MCV (MCV2) at village and township levels. *OR* odds ratio, *CI* confidence interval

At the village level, the absence of village doctors in routine vaccination services (MCV1, *OR* = 1.85; MCV2, *OR* = 2.11) negatively affected timely vaccination in children. At the township level, the vaccination appointment service (MCV1, *OR* = 2.96; MCV2, *OR* = 2.74), vaccination services on non-market days (MCV1, *OR* = 2.48), and insufficient and clustered routine vaccination sessions (MCV1, *OR* = 2.08) were significant barriers for children to receive timely vaccination (Fig. 2).

## Discussion

The findings revealed that children were less likely to receive timely measles vaccinations if their primary guardians had poor vaccination knowledge, weak vaccine confidence, few practices towards vaccination, or a low degree of satisfaction with the vaccination service. This is similarly observed in children whose village doctors did not participate in the routine vaccination service or whose township hospital did not provide monthly vaccination notice sheets, did not make formal vaccination appointments, did not provide sufficient and uniformly distributed sessions for routine vaccination, or did not offer vaccination services on open country market days.

The aforementioned factors may have complex effects on the vaccination status of children. First, several children were left in the care of their grandparents or other relatives in their rural hometowns. It is widely acknowledged that an intact family plays a critical role in child-rearing, and parental absences from labour migration pose potential threats to the psychological well-being and physical health of the child, including timely vaccination [25]. A previous study in rural China revealed that after adjusting for family socio-economic status indicators, such children were significantly more likely to receive untimely vaccination for MCV1 than children with intact families (*OR* = 1.33, 95% *CI *1.02–1.75) [26]. Second, most primary guardians had lower education attainment and worked as farmers, which may be associated with poor vaccination confidence and knowledge regarding timely vaccination. A previous study that integrated the protection motivation theory model, health belief model, and theory of planned behaviours found that children whose guardians had traditional misconceptions and poor knowledge about measles, measles vaccine, and immunisation schedules were more likely to be vaccinated late or non-vaccinated [27]. Third, older guardians are preoccupied with daily agricultural livelihood and may be unaware of the importance of timely vaccination for the children, thus resulting in few positive practices towards measles vaccination [28].

In addition, the transformation of health system services in rural China may also affect vaccination services. The updated health service system requires the provision of vaccination services at township hospitals [29]. However, these services are offered on a time-fixed and location-listed system. With this current policy, rural children rely solely on the initiative of their guardians to be vaccinated on time. Additionally, a lack of provision for active family vaccination services from township hospitals might negatively affect timely vaccination [30]. Our findings contradict those of previous studies in Kenya and Burkina Faso, where rural children had the advantage of timely vaccination over urban children, as a door-to-door vaccination service was regularly supplied by mobile vaccination teams in rural areas [31].

Furthermore, the centralised vaccination mode was not ideal. Specifically, the allocation of human health resources was largely unmet, regular sessions for routine vaccination were insufficient, vaccination notice and appointment services were not adequately provided, and involvement of village doctors in routine vaccination services was not required. Insufficient sessions for routine vaccination and inadequate vaccination appointment services might be indirectly associated with human health resources. Previous studies in Ethiopia, Turkey, and China revealed that shortages of health human resources were barriers to the accessibility of primary health care services in rural areas, including routine immunisation services [32–34]. Thus, non-ideal vaccination services considerably reduce the timeliness of vaccination in children.

Lastly, Guangxi is an underdeveloped mountainous region, where the public transportation system is poor and travel to vaccination clinics is difficult across villages. The vaccination service on non-market days and outreach vaccination services by the township hospitals remained unimplemented in most counties, thereby preventing guardians from arranging timely vaccinations for children in their care [35]. Due to the joint effect of the primary guardian’s discontent and their KABP and the unideal vaccination services, children were at a higher risk of being vaccinated late or not at all, particularly children without intact families.

The strengths of this study should be noted. This study systematically explored the determinants of untimely measles vaccination at the household, village clinic, and township hospital levels. The findings in this study gain a comprehensive insight into vaccination-related health system barriers to the timeliness of vaccination.

The limitations of this study need to be acknowledged. First, socio-economic status indicators were not included in the model, although other studies have revealed the effect of these variables on untimely vaccination in children. Compared with the KABP of primary guardians, socio-economic status indicators might be proxy variables. Second, the study did not further compare the differences in findings between the village-household, hospital-household, and hospital-village-household models; therefore, this was not a complete analysis. Third, we did not fit multivariate multilevel logistic regression models to further identify determinants of timely and complete measles vaccination, as vaccination status of MCV1 and MCV2 might be intercorrelated. Fourth, the importance, causation pathway, and inter-relationship among significant multilevel determinants were not explored in this study; thus, causal relationships could not be determined. Further studies should be conducted to understand the web of cause among determinants using directed acyclic graphs, Bayesian network models, and multilevel structural equation models [36]. Lastly, sampling weights of cluster surveys were not taken into consideration in the multilevel models, as no statistical approach is yet available to realise this analysis.

### Policy implications

Although the survey was conducted in 2015, the vaccination service modes and policies of routine vaccination have not been changed at vaccination agencies since then. Furthermore, the timeliness and completeness of routine vaccination are not highly put on the agenda of vaccination service agencies. Therefore, our findings still have valuable implications for vaccination service agencies to improve the quality of routine vaccination services. Findings may serve as a guide for policymakers and vaccination practitioners in the optimisation of the present vaccination policy and the improvement of the timely MCV administration in rural China as well as in developing countries in a similar context.

It was determined that at the household level, primary guardians had poor vaccination knowledge, which may be corrected by local health departments publicising vaccination-related knowledge across multiple platforms to improve awareness. At the village level, village doctors act as messengers of vaccination services; thus, they should be motivated to take responsibility for vaccination education, mobilisation, and promotion. Moreover, they should inform primary guardians about vaccination dates by distributing vaccination notices or by using other reminders.

Furthermore, given the disparity between the overburdened workload and the unmet health human resource allocation in township hospitals, full-time vaccination workgroups should be established independently from the essential public health sectors. Due to the inconvenient local public transportation, which affects visits to township hospitals, it is suggested that vaccination sessions be fixed on country market days. Considering the limited and clustered sessions for routine vaccination, the extension and uniform distribution of the sessions within a month are recommended. Coupled with the poor transportation system and weak awareness of timely vaccination among primary guardians, outreach vaccination services should be provided by mobile vaccination teams in remote villages to reduce the spatial inequity and provide access to timely vaccination services. Most primary guardians of these children were found to be older, less educated, and more preoccupied with agricultural livelihood; thus, formal (written) appointments on the next vaccination dates and monthly vaccination notices should be arranged for the guardians. In addition, children who are not vaccinated on time should be followed up individually through calls, and supplementary vaccination campaigns may be necessary for children with incomplete or untimely vaccination.

## Conclusions

Factors associated with untimely measles vaccination among children in rural Guangxi include having primary guardians with poor knowledge, weak vaccine confidence, and few practices towards measles vaccination; the absence of village doctors in routine vaccination services; and inconvenient vaccination services in township hospitals. Priority measures targeting improvement of these factors as well as removal of vaccination-related health system barriers should be urgently implemented to achieve timely measles vaccinations in rural China.

## Supplementary Information

**Additional file 1. Appendix S1:** The location of Guangxi in China. **Appendix S2:** Structured questionnaire at household level. **Appendix S3:** Semi-structured questionnaire at village level. **Appendix S4:** Semi-structured questionnaire at township level. **Appendix S5:** Measurement of independent variables. **Appendix S6:** Parameter estimation for null models at different levels.

## Data Availability

The datasets used and/or analyzed during the current study are available from the corresponding author on reasonable request.

## Abbreviations

ASEAN: The Association of Southeast Asian Nations
MCV: Measles-containing vaccine
MCV1: The first dose of measles-containing vaccine
MCV2: The second dose of measles-containing vaccine
*OR*: Odds ratio
WHO/EUP: World Health Organization Europe Region
WHO/WPR: WHO Western Pacific Region
