# Supplementary material for: Multi-level determinants of failure to receive timely and complete measles vaccinations in Southwest China: a mixed methods study
Source: Infect Dis Poverty. 2021 Jul 22;10:102. doi: 10.1186/s40249-021-00885-6 (PMC8296749; doi:10.1186/s40249-021-00885-6)
Supplement: Supplementary file 1 — Additional file 1. Appendix S1: The location of Guangxi in China. Appendix S2: Structured questionnaire at household level. Appendix S3: Semi-structured questionnaire at village level. Appendix S4: Semi-structured questionnaire at township level. Appendix S5: Measurement of independent variables. Appendix S6: Parameter estimation for null models at different levels. [file 40249_2021_885_MOESM1_ESM.docx]

**[Additional file 1](https://static-content.springer.com/esm/art%3A10.1186%2Fs40249-018-0512-6/MediaObjects/40249_2018_512_MOESM1_ESM.pdf)**

**Appendix S1 The location of Guangxi in China**

**
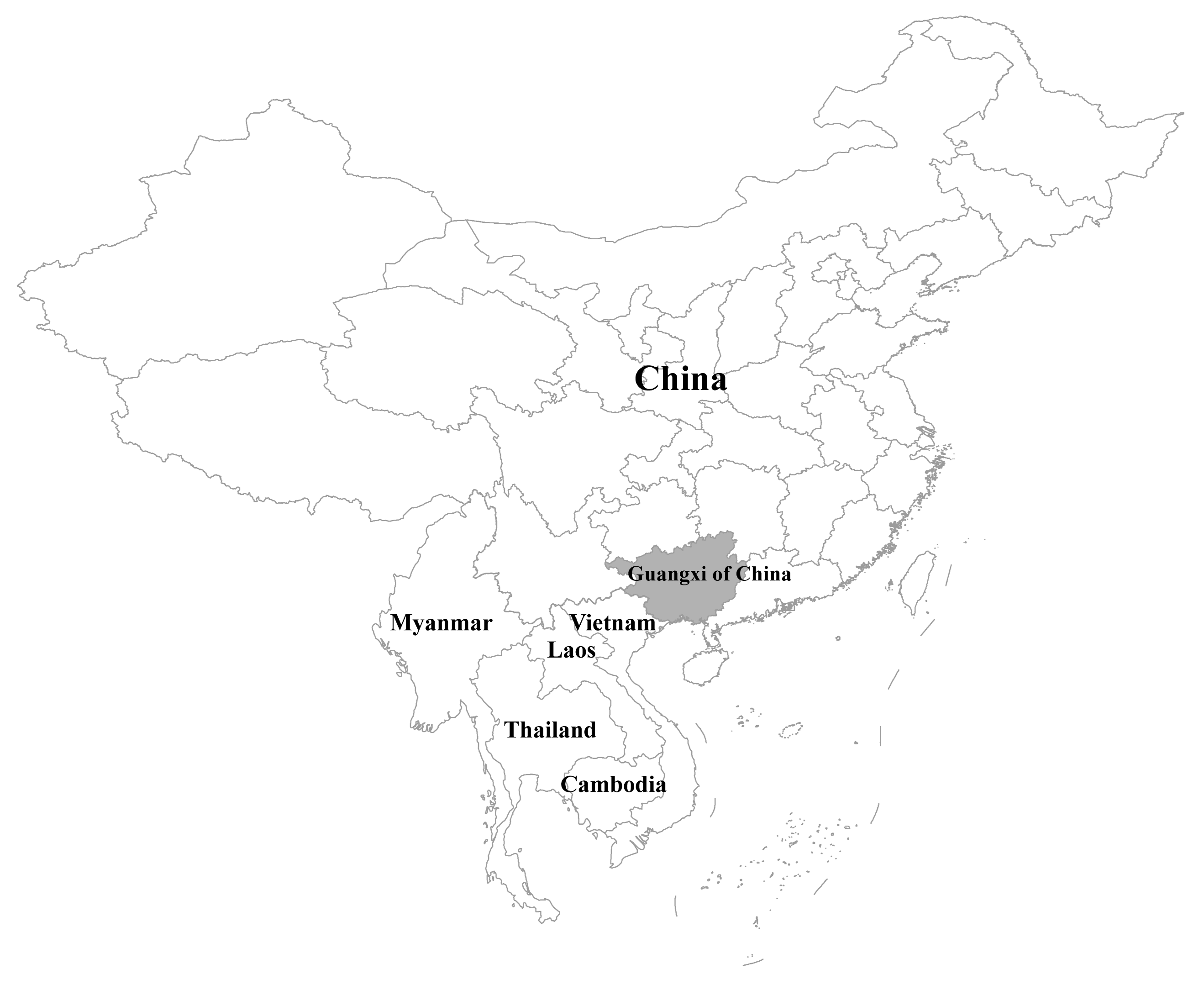
**

**Appendix S2 Structured questionnaire at household level**

| Questionnaire ID: (stamp number)  Data entry round:[ ]1. [ ] 2. |
| --- |

| Date___/____/____ [ ][ ]/[ ][ ]/[ ][ ][ ][ ] |
| --- |
| Start time of interview (system 24 h) [ ][ ]:[ ][ ] |
| County [ ] |
| Town [ ] |
| Village No. [ ][ ][ ] |
| Name of householder ___________ |
| Geographic environment of household [ ]0.moutians [ ]1. hills |
| Meet eligibility criteria [ ]0.No [ ]1.Yes |
| Permission for interview [ ]0.No [ ]1.Yes |

**Part I : Socio-demographic and socio-economic information**

| 1. Target child’s name _________ |
| --- |
| 2. Child’ gender [ ]0.female [ ]1.male |
| 3. Child’ race [ ]1.Han [ ]2.Zhuang [ ]3.Yao [ ]4.Others (please specify)______________. |
| 4. Child’s birth date ___/___/201__ |
| 5.1 Birth order of target child  [ ]1.first [ ]2.second [ ]3. third or above |
| 6. Child’s birth place  [ ]1.government hospital [ ]2.private clinics [ ]3.home [ ]4. others (please specify)______ |
| 7. Does birth of the child correspond to family planning strategy?  [ ]0.No [ ]1.Yes |
| 8. Is the child registered in the household system?  [ ]0. No [ ]1.Yes |
| 9. Does the child have health insurance scheme?  [ ]0.No [ ]1.Yes |
| 10. No. of children in householder? [ ]children |
| 11. Mean interval between pregnancies for target child’ mother.  [ ]1. one year [ ]2.two years [ ]3.three years or above |
| 12. Name of primary guardian ______ |
| 13. Gender of primary guardian  [ ]0.female [ ]1.male |
| 14. Age of primary guardian [ ][ ]years old |
| 15. Race of primary guardian  [ ]1.Han [ ]2.Zhuang [ ]3.Yao [ ]4.Others (please specify)______________. |
| 16. The guardian’s education level  [ ]1.primary school or below [ ]2.junior middle school [ ]3.senior high school or technical secondary school [ ]4.college or above |
| 17 Occupation of guardian  [ ]1.peasant [ ]2.migrant worker [ ]3.clerk [ ]4.private owner [ ]5.others (please specify)____________. |
| 18. Guardian is the child’s  [ ]1.parents [ ]2.grandparents [ ]3.other relatives (please specify) _______ |
| 19. No. of persons in your household [ ][ ]persons |
| 20. Family annual income [ ][ ][ ][ ][ ]CNY |
| 21. Age of child’s father [ ][ ]years old |
| 22. Education level of child’s father  [ ]1.primary school or below [ ]2.junior middle school [ ]3.senior high school or technical secondary school [ ]4.college or above |
| 23. Occupation of child’s father  [ ]1.peasant [ ]2.migrant worker [ ]3.clerk [ ]4.private owner [ ]5.others (please specify)____________. |
| 24.Annual average working time outside for father  [ ]1.never working outside [ ]2.1-3months  [ ]3. 4-6months [ ]4. 7-9months [ ]5.10-12months |
| 25. Marital status of child’s father  [ ]1.unmarried [ ]2. married [ ]3.divorced [ ]4.widowed |
| 26. Age of child’s mother [ ][ ]years old |
| 27. Education level of child’s mother  [ ]1.primary school or below [ ]2.junior middle school [ ]3.senior high school or technical secondary school [ ]4.college or above |
| 28. Occupation of child’s mother  [ ]1.peasant [ ]2.migrant worker [ ]3.clerk [ ]4.private owner [ ]5.others (please specify)____________. |
| 29. Annual average working time outside for mother  [ ]1.never working outside [ ]2.1-3months  [ ]3. 4-6months [ ]4. 7-9months [ ]5.10-12months |
| 30. Marital status of child’s mother  [ ]1.unmarried [ ]2. married [ ]3.divorced [ ]4.widowed |
| 31. Is the child’s mother local people?  [ ]0.No [ ]1.Yes |
| 32. Did child’ mother have previous history of measles?  [ ]0.No [ ]1.Yes [ ]2. Unknown |
| 33. Did child’s mother receive measles vaccination before?  [ ]0.No [ ]1.Yes [ ]2. Unknown |
| 34. Has the child gotten measles already?  [ ]0.No [ ]1.Yes [ ]2. Unknown |

**Part II : Measles vaccination information**

| 1. Does the child have a vaccination certificate?  [ ]0.No [ ]1.Yes |
| --- |
| 2. Does the child have a vaccination card?  [ ]0.No [ ]1.Yes |
| 3. Has the child received the first dose of measles vaccine?  [ ]0.No [ ]1.Yes |
| 4. Date of receiving the first dose was ___/___/201__  [ ][ ]/[ ][ ]/[2][0][1][ ] |
| 5. Vaccination status of first dose according to the recommended schedule (judged and filled by interviewer)  [ ]1.non-vaccinated [ ]2.early [ ]3.delayed [ ]4.timely |
| 6. Place of receiving 1^st^ dose  [ ]1.governmental hospital [ ]2.township health centre [ ]3.village clinic [ ]4.private clinics |
| 7. Was there a payment for 1^st^ dose?  [ ]0.No [ ]1.Yes [ ]2.Unknown |
| 8. Has the child received the second dose of measles vaccine?  [ ]0.No [ ]1.Yes |
| 9. Date of receiving the second dose was ___/___/201__  [ ][ ]/[ ][ ]/[2][0][1][ ] |
| 10. Vaccination status of second dose according to the recommended schedule (judged and filled by interviewer)  [ ]1. non-vaccinated [ ]2. early [ ]3.delayed [ ]4.timely |
| 11. Place of receiving 2^nd^ dose  [ ]1.governmental hospital [ ]2.township health centre [ ]3.village clinic [ ]4.private clinics |
| 12. Was there a payment for 2^nd^ dose?  [ ]0.No [ ]1.Yes [ ]2.Unknown |
| 13. Does guardian tell that the child received the 1st dose measles vaccine, despite without vaccination proof?  [ ]0.No [ ]1.Yes |
| 14. Does guardian tell that the child received the 2nd dose measles vaccine, despite without vaccination proof?  [ ]0.No [ ]1.Yes |
| 15. Reasons for failure to receive the first dose measles vaccine during the recommended schedule.(multiple choices)  15.1 It is better to receive 1^st^ dose early  [ ]0.No [ ]1.Yes  15.2 Doctor suggested receiving 1^st^ dose early  [ ]0.No [ ]1.Yes  15.3 It is not issue to receive in a few days advance  [ ]0.No [ ]1.Yes  15.4 It is better to be vaccinated naturally. [ ]0.No [ ]1.Yes  15.5 In fact, the guardian known vaccination, but he was unaware of vaccination schedule  [ ]0.No [ ]1.Yes  15.6 Lack of immunization formation  [ ]0.No [ ]1.Yes  15.7 Lack of awareness of vaccination  [ ]0.No [ ]1.Yes  15.8 Fears of side effects  [ ]0.No [ ]1.Yes  15.9 Wrong ideas about contraindications  [ ]0.No [ ]1.Yes  15.10 No faith in immunization  [ ]0.No [ ]1.Yes  15.11 Have no time to take child to township hospital  [ ]0.No [ ]1.Yes  15.12 It is inconvenience travel to township hospital  [ ]0.No [ ]1.Yes  15.13 The time for immunization is inconvenient for me  [ ]0.No [ ]1.Yes  15.14 Vaccinator was absent  [ ]0.No [ ]1.Yes  15.15 Measles vaccine was unavailable  [ ]0.No [ ]1.Yes  15.16 child ill  [ ]0.No [ ]1.Yes  15.17 Child moved to other place  [ ]0.No [ ]1.Yes  15.18 The waiting time for vaccination in clinics is long  [ ]0.No [ ]1.Yes  15.19 Payment for vaccine  [ ]0.No [ ]1.Yes  15.20 Not registering for child  [ ]0.No [ ]1.Yes  15.20 Others, please specify____________ |
| 16. Reasons for failure to receive the 2^nd^ dose measles vaccine during the recommended schedule.(multiple choices)  16.1 Thinking receive 1^st^ dose is enough  [ ]0.No [ ]1.Yes  16.2 The schedule is so long that we think the child still have time to receive measles vaccine  [ ]0.No [ ]1.Yes  16.3 Lack of immunization formation  [ ]0.No [ ]1.Yes  16.4 Lack of awareness of vaccination  [ ]0.No [ ]1.Yes  16.5 Fears of side effects  [ ]0.No [ ]1.Yes  16.6 Wrong ideas about contraindications  [ ]0.No [ ]1.Yes  16.7 No faith in immunization  [ ]0.No [ ]1.Yes  16.8 Have no time to take child to township hospital  [ ]0.No [ ]1.Yes  16.9 It is inconvenience travel to township hospital  [ ]0.No [ ]1.Yes  16.10 The time for immunization is inconvenient for me  [ ]0.No [ ]1.Yes  16.11 Vaccinator was absent  [ ]0.No [ ]1.Yes  16.12 Measles vaccine was unavailable  [ ]0.No [ ]1.Yes  16.13 child ill  [ ]0.No [ ]1.Yes  16.14 Child moved to other place  [ ]0.No [ ]1.Yes  16.15 The waiting time for vaccination in clinics is long  [ ]0.No [ ]1.Yes  16.16 Payment for vaccine  [ ]0.No [ ]1.Yes  16.17 Not registering for child  [ ]0.No [ ]1.Yes  16.18 Others, please specify____________ |
| 17. Have you received a vaccination notice?  [ ]0.No [ ]1.Unknown [ ]2.Yes |
| 18. How did the healthcare workers inform you about vaccination? By  [ ]1.none [ ]2.appointment [ ]3.telephone [ ]4.text messages [ ]5.home service [ ]6.bullentin [ ]7.broadcast [ ]8.letter [ ]9.others, please specify_________ |
| 19. Did the healthcare workers hold a pre-vaccination physical examination for your child?  [ ]0.No [ ]1.Unknown [ ]2.Yes |
| 20. Did the healthcare workers explain to you vaccination information including indications, contraindication, functions and side effects of measles vaccine.  [ ]0.No [ ]1.Unknown [ ]2.Yes |
| 21. Have you been given post vaccination advice?  [ ]0.No [ ]1.Unknown [ ]2.Yes |

**Part III: Guardian’s knowledge of measles vaccination**

| 1. Do you know the address for the place of vaccination?  [ ]0.No [ ]1.Yes, please specify___________ |
| --- |
| 2. Do you think vaccination certificate should be checked when child is enrolled into nursery or kindergarten?  [ ]0.No [ ]1.Unknown [ ]2.Yes |
| 3. Do you think “sugar pill” could be taken with hot water or milk? [ ]0.No [ ]1.Unknown [ ]2.Yes |
| 4. The sugar pill is actually a vaccine against  [ ]1.tuberculosis [ ]2.poliomyelitis [ ]3.tetanus, diphtheria and pertussis [ ]4. measles |
| 5. BCG vaccine is against  [ ]1.tuberculosis [ ]2.poliomyelitis [ ]3.tetanus, diphtheria and pertussis [ ]4. measles |
| 6. Is measles an infectious disease?  [ ]0.No [ ]1.Unknown [ ]2.Yes |
| 7. Do you think measles vaccine is covered under EPI?  [ ]0.No [ ]1.Unknown [ ]2.Yes |
| 8. MMR is against  [ ]1.tuberculosis [ ]2.poliomyelitis [ ]3.tetanus, diphtheria and pertussis [ ]4. measles |
| 9. Do you think measles immunization is necessary to your child?  [ ]0.No [ ]1.Unknown [ ]2.Yes |
| 10. Do you think measles vaccines are charged?  [ ]0.No [ ]1.Unknown [ ]2.Yes |
| 11. Do you think child measles immunization service is freely available for migrant children?  [ ]0.No [ ]1.Unknown [ ]2.Yes |
| 12. Do you think that in case of fever, allergy and acute or chronic disease, the child could still receive measles vaccine?  [ ]0.No [ ]1.Unknown [ ]2.Yes |
| 13. How many doses are needed for measles vaccination within two years old?  [ ]1. one dose [ ]2.two doses [ ]3.three doses |
| 14. Do you think it is normal to have mild adverse reactions such as fever after measles vaccination?  [ ]0.No [ ]1.Unknown [ ]2.Yes |
| 15. Do you think measles vaccination is related to illness after vaccination? [ ]0.No [ ]1.Unknown [ ]2.Yes |
| 16. The first dose of measles vaccine is received when child is ____months old  [ ]1.six months [ ]2.eight months [ ]3. nine months [ ]4.twelves months |
| 17. Do you think it is necessary that a child is observed for at least 30 minutes after measles vaccination?  [ ]0.No [ ]1.Unknown [ ]2.Yes |

**Part IV: Guardian’s attitude and health belief towards measles vaccination**

**Perception of susceptibility to measles**

| 1. Measles is an infectious disease and children are the susceptible population  [ ]1.strongly disagree [ ]2.disagree [ ]3.not sure [ ]4.agree [ ]5.strongly agree |
| --- |
| 2.Without vaccination, it is likely that children will catch measles  [ ]1.strongly disagree [ ]2.disagree [ ]3.not sure [ ]4.agree [ ]5.strongly agree |
| 3.Without timely vaccination, children are more likely to catch measles  [ ]1.strongly disagree [ ]2.disagree [ ]3.not sure [ ]4.agree [ ]5.strongly agree |
| 4.Without completing two doses of measles vaccines within two years old, children are more likely to catch measles  [ ]1.strongly disagree [ ]2.disagree [ ]3.not sure [ ]4.agree [ ]5.strongly agree |
| 5. Without timely and complete measles vaccination, it is more likely that children will have complications of measles, e.g. pneumonia  [ ]1.strongly disagree [ ]2.disagree [ ]3.not sure [ ]4.agree [ ]5.strongly agree |

**Perception of severity in measles**

| 1. Measles is a serious illness  [ ]1.strongly disagree [ ]2.disagree [ ]3.not sure [ ]4.agree [ ]5.strongly agree |
| --- |
| 2. Measles is one of the major causes of death among infants  [ ]1.strongly disagree [ ]2.disagree [ ]3.not sure [ ]4.agree [ ]5.strongly agree |
| 3. Measles will serious negative impacts on child’s health status, daily life and study.  [ ]1.strongly disagree [ ]2.disagree [ ]3.not sure [ ]4.agree [ ]5.strongly agree |
| 4. Measles will considerably increase the socio-economic burden in your family  [ ]1.strongly disagree [ ]2.disagree [ ]3.not sure [ ]4.agree [ ]5.strongly agree |

**Perception of benefit from measles vaccination**

| 1. Vaccination will protect my child against measles  [ ]1.strongly disagree [ ]2.disagree [ ]3.not sure [ ]4.agree [ ]5.strongly agree |
| --- |
| 2. Vaccination will alleviate symptoms of measles.  [ ]1.strongly disagree [ ]2.disagree [ ]3.not sure [ ]4.agree [ ]5.strongly agree |
| 3. Vaccination will stop the transmission of measles among children  [ ]1.strongly disagree [ ]2.disagree [ ]3.not sure [ ]4.agree [ ]5.strongly agree |
| 4. Timely vaccination will increase the herd immunity against measles  [ ]1.strongly disagree [ ]2.disagree [ ]3.not sure [ ]4.agree [ ]5.strongly agree |

**Perception of barriers to measles vaccination**

| 1. MMR has serious side effects  [ ]1.strongly disagree [ ]2.disagree [ ]3.not sure [ ]4.agree [ ]5.strongly agree |
| --- |
| 2. It is better to get natural immunity by catching measles  [ ]1.strongly disagree [ ]2.disagree [ ]3.not sure [ ]4.agree [ ]5.strongly agree |
| 3. MMR is too much for my child’s body to cope with, so I’d prefer to the single measles shot.  [ ]1.strongly disagree [ ]2.disagree [ ]3.not sure [ ]4.agree [ ]5.strongly agree |
| 4. Time and transportation to township hospital makes it hard to take my child for vaccination  [ ]1.strongly disagree [ ]2.disagree [ ]3.not sure [ ]4.agree [ ]5.strongly agree |
| 5. Child can still catch measles again in spite of vaccination  [ ]1.strongly disagree [ ]2.disagree [ ]3.not sure [ ]4.agree [ ]5.strongly agree |
| 6. I do not know the vaccination dates when my child needs to get vaccination.  [ ]1.strongly disagree [ ]2.disagree [ ]3.not sure [ ]4.agree [ ]5.strongly agree |
| 7. Township hospital will charge me for my child’s vaccination  [ ]1.strongly disagree [ ]2.disagree [ ]3.not sure [ ]4.agree [ ]5.strongly agree |
| 8. My child is allergic to vaccines or eggs.  [ ]1.strongly disagree [ ]2.disagree [ ]3.not sure [ ]4.agree [ ]5.strongly agree |

**Perception of cues to action**

| 1. Healthcare workers advised my children to take MMR  [ ]1.no [ ]2.not sure [ ]3.yes |
| --- |
| 2. Relatives suggested me to vaccinate my child  [ ]1.no [ ]2.not sure [ ]3.yes |
| 3. I read some brochures on vaccination  [ ]1.no [ ]2.not sure [ ]3.yes |
| 4. I saw or heard of measles case in my village or surrounding areas  [ ]1.no [ ]2.not sure [ ]3.yes |
| 5. I was aware of the vaccination dates in child’s vaccination certificate, arranged by vaccination professionals  [ ]1.no [ ]2.not sure [ ]3.yes |

**Perception of self efficacy**

| 1. Are you willing to let children completely participate in the national immunization programme?  [ ]1.strongly unwilling [ ]2.unwilling [ ]3.not sure [ ]4.willing [ ]5.strongly willing |
| --- |
| 2. Are you willing to make child vaccinated on time?  [ ]1.strongly unwilling [ ]2.unwilling [ ]3.not sure [ ]4.willing [ ]5.strongly willing |
| 3. Are you willing to pay for a measles vaccination?  [ ]1.strongly unwilling [ ]2.unwilling [ ]3.not sure [ ]4.willing [ ]5.strongly willing |

**Part V: Guardian’s practice towards measles vaccination**

| 1. Have you taken advises on measles immunization from others?  [ ]1.No, I haven’t [ ] Not sure [ ]3.Yes, I have |
| --- |
| 2. Have you taken your child to vaccinate complying with the vaccination notice?  [ ]1.No, I haven’t [ ] Not sure [ ]3.Yes, I have |
| 3. Have you made appointment with doctors on the next vaccination date when your child has finished the present vaccine?  [ ]1.No, I haven’t [ ] Not sure [ ]3.Yes, I have |
| 4. Without vaccination notice, have you ever actively taken your child to township hospital for vaccination?  [ ]1.No, I haven’t [ ] Not sure [ ]3.Yes, I have |
| 5. Have you ever actively been township hospitals to consult vaccination information, such as safety, effect and time?  [ ]1.No, I haven’t [ ] Not sure [ ]3.Yes, I have |

**Part VI: Guardian’s satisfaction with measles vaccination service**

| 1. How do you feel about the travel distance between home and place of vaccination?  [ ]1.very unsatisfied [ ]2.somewhat unsatisfied [ ]3.neutral [ ]4.somewhat satisfied [ ]5.very satisfied |
| --- |
| 2. How do you feel about professional skills of doctor and nurse at the place of vaccination?[ ]1.very unsatisfied [ ]2.somewhat unsatisfied [ ]3.neutral [ ]4.somewhat satisfied [ ]5.very satisfied |
| 3. How do you feel about introductions of immunization at the place of vaccination?  [ ]1.very unsatisfied [ ]2.somewhat unsatisfied [ ]3.neutral [ ]4.somewhat satisfied [ ]5.very satisfied |
| 4. How do you feel about the way the notice of vaccination to be sent?  [ ]1.very unsatisfied [ ]2.somewhat unsatisfied [ ]3.neutral [ ]4.somewhat satisfied [ ]5.very satisfied |
| 5. How do you feel about the pre-vaccination physical examination?  [ ]1.very unsatisfied [ ]2.somewhat unsatisfied [ ]3.neutral [ ]4.somewhat satisfied [ ]5.very satisfied |
| 6. How do you feel about the post-vaccination parent advice being told by the doctor or nurse?  [ ]1.very unsatisfied [ ]2.somewhat unsatisfied [ ]3.neutral  [ ]4.somewhat satisfied [ ]5.very satisfied |
| 7. How do you feel about the doctor or nurse’s familiarity with your child?  [ ]1.very unsatisfied [ ]2.somewhat unsatisfied [ ]3.neutral [ ]4.somewhat satisfied [ ]5.very satisfied |
| 8. How do you feel about the doctor or nurse’s answer and attitude to your questions?  [ ]1.very unsatisfied [ ]2.somewhat unsatisfied [ ]3.neutral [ ]4.somewhat satisfied [ ]5.very satisfied |
| 9. How do you feel about sanitary conditions in place of vaccination?  [ ]1.very unsatisfied [ ]2.somewhat unsatisfied [ ]3.neutral [ ]4.somewhat satisfied [ ]5.very satisfied |
| 10. How do you feel about promotion of EPI performed by village doctor?  [ ]1.very unsatisfied [ ]2.somewhat unsatisfied [ ]3.neutral [ ]4.somewhat satisfied [ ]5.very satisfied |
| 11. How do you feel about service appointment for vaccination?  [ ]1.very unsatisfied [ ]2.somewhat unsatisfied [ ]3.neutral [ ]4.somewhat satisfied [ ]5.very satisfied |
| 12. How do you feel about spending time in waiting for vaccination?  [ ]1.very unsatisfied [ ]2.somewhat unsatisfied [ ]3.neutral [ ]4.somewhat satisfied [ ]5.very satisfied |

**Appendix S3 Semi-structured questionnaire at village level**

| Questionnaire ID: (stamp number)  Data entry round:[ ]1. [ ] 2. |
| --- |

| Date___/____/_____ [ ][ ]/[ ][ ]/[ ][ ][ ][ ] |
| --- |
| Start time of interview (system 24 h) [ ][ ]:[ ][ ] |
| County [ ] |
| Town [ ] |
| Village No. [ ][ ][ ] |
| Coordinates of village(measured by interviewer, via GPS)  Latitude [ ][ ].[ ][ ]  Longitude [ ][ ][ ].[ ][ ]  Travel-time to township hospital minutes  Travel-distance to township hospital km |
| Name of village doctor ___________ |
| Meet eligibility criteria [ ]0.No [ ]1.Yes |
| Permission for interview [ ]0.No [ ]1.Yes |
| Having measles cases in the past three years  [ ]0. No [ ]1. Yes |

**Part I: Allocation of vaccination-related health resource**

| 1.1 No. of doctors in your village clinic [ ]person  1.2 The population size in your village ______residents |
| --- |
| 2. Sociodemographic characteristics of 1^st^ doctor  2.1 Age [ ][ ]  2.2 Sex [ ]0.female [ ]1.male  2.3 Education level  [ ]1.primary school or below [ ]2.junior middle school [ ]3.senior high school or technical secondary school [ ]4.college or above  2.4 Professional title [ ]1.no title [ ]2. junior professional title [ ]3. intermediate professional title  2.5 The length of service in immunization [ ][ ]years |
| 3. Sociodemographic characteristics of 2^nd^ doctor  3.1 Age [ ][ ]  3.2 Sex [ ]0.female [ ]1.male  3.3 Education level  [ ]1.primary school or below [ ]2.junior middle school [ ]3.senior high school or technical secondary school [ ]4.college or above  3.4 Professional title [ ]1.no title [ ]2. junior professional title [ ]3. intermediate professional title  3.5 The length of service in immunization [ ][ ]years |
| 4.1 Does the village clinic you working for own any autoclave? [ ]0.No [ ]1.Yes  4.2 Do you prescribe drugs or use injections for residents?  [ ]0. No [ ]1. Yes |
| 5.1 Does the village clinic you working for own any refrigerator or device for vaccine storage?  [ ]0.No [ ]1.Yes |
| 6.1 Is your clinic responsible for vaccination service?  [ ]0.No [ ]1.Yes  6.2 Can the children get home vaccination service in village?  6.2 Do you involve in the routine vaccination service? What do you do in that activity?  6.3 Do you participate in the supplementary immunization activities or catch-up vaccination campaigns? |
| 7.1 Does your clinic get financial subsidy for vaccination service? [ ]0.No [ ]1.Yes  7.2 How much specific subsidy per month do you get from the vaccination service?  7.3 How much subsidy per month do you get from the national essential public health services?  7.4 How much income do you have through providing fee-to-pay health services? |
| 8. Do you feel satisfied with the financial subsidy in immunization?  [ ]1.very unsatisfied [ ]2.somewhat unsatisfied [ ]3.neutral [ ]4.somewhat satisfied [ ]5.very satisfied |
| 9. Regarding the amount and timeliness of financial subsidy for EPI, how do you perceive? |
| 10. In light of the allocation of health human resource (e.g. quantity and quality of village doctors, education level, competence in vaccination service), how do you think about? |

**Part II: Vaccination provider-related factors (Involvement in vaccination service)**

| 1.1 Do you participate in vaccination promotion currently?  [ ]1.never [ ]2.sometimes [ ]3.often [ ]always  1.2 How do you mobilize village residents to vaccinate?  1.3 How do you perceive the role of village doctor in vaccination service? |
| --- |
| 2. Do you participate in management of age-appropriate children for immunization currently?  [ ]1.never [ ]2.sometimes [ ]3.often [ ]always |
| 3. Is your work in immunization a concurrent post?  [ ]0.No [ ]1.Yes |
| 4. How do you think your attitude towards vaccination service?  [ ]1.very negative [ ]2.negative [ ]3.neutral [ ]4.postive [ ]5.very positive |
| 5. Would you please describe how well you provide vaccination service? How well do you enjoy in this activity? |
| 6. In light of vaccination service, how do you think about your sense of responsibility? |
| 7.1 Are you familiar with the basic information about target population for childhood immunization in your village?  [ ]1.not at all [ ]2.not very [ ]3.neither [ ]4.familiar [ ]5.very familiar  7.2 How can you manage the target immunization children in your village? |
| 8. In your opinion, are you competent with vaccination service?  [ ]1.very incompetent [ ]2. incompetent [ ]3.fair [ ]4. competent [ ]5.very competent |
| 9. Regarding the requirement of knowledge in vaccination service, how do you assess your own knowledge? |
| 10.1 How do you inform child’s guardian about vaccination?  [ ]1. none [ ]2.vaccination notice sheet [ ]3.telephone notice [ ]4. short message service [ ]5.home service [ ]6.bulletin [ ]7.broadcast [ ]8.sending a message to someone else [ ]9.others, please specify____________.  10.2 What vaccination information do you provide for guardians? (vaccination dates, vaccine doses, requirement, etc.) |
| 11. Do village headers participate in vaccination mobilization or promotion?  [ ]0.No [ ]1.Yes |
| 12. How many times per year do healthcare workers in township hospital evaluate you performance? |

**Part III: Perception of the current vaccination policy**

| 1. Do you familiar with the current vaccination mode? [ ]0.No [ ]1.Yes |
| --- |
| 2.1 Would you please introduce briefly the current vaccination mode? (centralized in township hospital or decentralized in village clinics, providing vaccination services on market days or non-market days, outreach service in villages)  2.2 How about the frequency of routine vaccination session? Do the regular sessions uniformly distribute over a month?  2.3 How about the frequency of catch-up vaccination session? |
| 3. How do you perceive the effect of current vaccination mode in rural areas?  (strength and weakness) |
| 4. How do you think about the encouragement and incentives of vaccination policy for vaccination professionals in rural areas? |
| 5. How do you see the degree of village doctor’s participation in vaccination service? |
| 6. How do you think the attitudes of higher health authorities towards vaccination service? |
| 7. Compared with the decentralized vaccination mode provided by village doctors, what is the strength and weakness of the centralized vaccination mode in rural areas? |
| 8. To make it well-run in rural areas, what do you suggest to optimize or improve the current vaccination policy?  (health human resource allocation, financial subsidy, incentives, village doctor’s role in vaccination service, regular sessions for routine vaccination service, vaccination dates, etc ) |

**Appendix S4**  **Semi-structured questionnaire at township level**

| Questionnaire ID: (stamp number)  Data entry round:[ ]1. [ ] 2. |
| --- |

| Date___/____/_____ [ ][ ]/[ ][ ]/[ ][ ][ ][ ] |
| --- |
| Start time of interview (system 24 h) [ ][ ]:[ ][ ] |
| County [ ] |
| Town [ ]  Minority ethnicity town[ ]  [ ]0. No [ ]1.Yes |
| Coordinates of township hospital (measured by interviewer, via GPS)  Latitude [ ][ ].[ ][ ]  Longitude [ ][ ][ ].[ ][ ]  Travel-time from hospital to the most far away village ____minutes  Travel-distance from hospital to the most far away village _____km |
| Name of doctor in township hospital ___________ |
| Meet eligibility criteria [ ]0.No [ ]1.Yes |
| Permission for interview [ ]0.No [ ]1.Yes |
| Having measles cases in the past three years  [ ]0. No [ ]1.Yes |

**Part I: Allocation of vaccination-related health resource**

| 1.1 No. of doctors responsible for vaccination service in your township hospital [ ][ ]person  1.2 No. of full-time healthcare workers responsible for vaccination service [ ] person  1.3 No. of part-time healthcare workers responsible for vaccination service [ ]person  1.4 Having full-time vaccination workgroup  [ ]0. No [ ]1.Yes  1.5 Population size in your town _______residents |
| --- |
| 2. Socio-demographic characteristics of vaccination healthcare workers in this township hospital:  proportion of age group; ration of male to female; proportion of education level; proportion of professional title, proportion of licensed (assistant) doctor, etc. |
| 3.1 No. of refrigerators for vaccine cold chain [ ][ ]refrigerators  3.2 No. of vehicles for vaccine cold chain [ ]vehicles  3.3 How do you think about the equipment of cold chain in your health centre? |
| 4. Number of affiliated township hospital, in addition to the vaccination center in[ ]hospital. [ ] clinics |
| 5. Taking the number of target children into account in your town, how do you think about the quantity of measles vaccine storage? |
| 6.1 Is there an EPI information system established in your town? [ ]0. No [ ]1.Yes  6.2 How do you think about the operation status and function of information system? |
| 7.1 Budget for cold chain per person-year  7.2 Perception of the budget for cold chain  7.3 Budget for vaccination service per person-year  7.4 Perception of the budget for vaccination service |
| 8.1 Salary of fiscal allotment for vaccination professional  8.2 Specific subsidy for vaccination service  8.3 Perception of the subsidy for vaccination service |
| 9. Regarding the amount and timeliness of government funding for EPI, how do you perceive? |
| 10. In light of the allocation of human health resource in your township hospital, how do you think about?  (the quantity and quality of vaccination professionals, education level, professional title, duration of working years, competence in vaccination service, full-time vaccination workgroup, etc.) |

**Part II: Vaccination provider-related factors (Involvement in vaccination service)**

| 1.1. Do you participate in vaccination mobilization currently?  [ ]1.never [ ]2.sometimes [ ]3.often [ ]4. always  1.2 Regarding vaccination, how do you mobilize guardians? |
| --- |
| 2.1. Do you participate in vaccination notice currently?  [ ]1.never [ ]2.sometimes [ ]3.often [ ]4. always  2.2. How do you inform them of vaccination?  [ ]1. none [ ]2.vaccinaion notice sheet [ ]3.telephone [ ]4.short message service [ ]5.home service [ ]6.bulletin [ ]7.broadcast [ ]8.sending a message to someone else [ ]9.others, please specify____________.  2.3 Do you prepare monthly vaccination notice for children?  [ ]0. No [ ]1.Yes |
| 3.1. Do you provide vaccination education for children and guardians currently?  [ ]1.never [ ]2.sometimes [ ]3.often [ ]4. always  3.2 What education do you provide for guardians? |
| 4.1 Do you provide appointment service for vaccination currently?  [ ]1.never [ ]2.sometimes [ ]3.often [ ]4. always  4.2 Do you provide a formal appointment sheet for children to remind of their vaccination dates? [ ]0.No [ ]1.Yes  4.3 Regarding vaccination service, how do you make appointment with guardians? |
| 5.1 Do you participate in the management of age-appropriate children for vaccination currently?  [ ]1.never [ ]2.sometimes [ ]3.often [ ]always  5.2 How do you manage village doctors, in term of vaccination service?  5.3 How do you perceive the role of village doctor in vaccination service?  5.4 How do you perceive the degree of village doctor’s participation in vaccination service |
| 6.1 How do you think your attitude towards vaccination service?  [ ]1.very negative [ ]2.negative [ ]3.neutral [ ]4.postive [ ]5.very positive  6.2 How do you perceive your attitudes? |
| 7. Would you please describe how well you provide vaccination service? How well do you enjoy in this activity? |
| 8. In light of vaccination service, how do you think about your sense of responsibility? |
| 9. 1 Are you competent with vaccination service?  [ ]1.very incompetent [ ]2. incompetent [ ]3.fair [ ]4. competent [ ]5.very competent  9.2 How do you perceive your competence? |
| 10. Regarding the requirement of knowledge in vaccination service, how do you view your own knowledge? |
| 11. Do village headers participate in vaccination mobilization or promotion?  [ ]0.No [ ]1.Yes |

**Part III: Performance of vaccination-related health system**

| 1.1 How many township hospital supervised by your health center?  1.2 How do you think about their roles in the vaccination health system of three-tier in rural areas? |
| --- |
| 2.1 How about the current vaccination service mode?  (centralized or decentralized vaccination service)  2.3 How about the frequency of routine vaccination session?  (sessions fixed on market days, sessions uniformly distributed over a month)  2.3 How about the frequency of catch-up vaccination sessions?  2.4 How about the outreach vaccination services in your town? |
| 3.1 How do you perceive the implementation effect of current vaccination mode in rural areas?  3.2 How do you perceive the difficulty in providing vaccination services?  (health human resources, finance funding for vaccination service, finance incentive for vaccination professionals, etc.)  3.3 What is the difficulty in maintaining running the cold chain? |

**Part IV: Perception of the current vaccination policy**

| 1. Would you please introduce briefly the current vaccination mode?  (centralized in township hospital or decentralized in village clinics, providing vaccination services on market days or non-market days, outreach service in villages) |
| --- |
| 2. Compared with the decentralized vaccination mode provided by village doctors, what is the strength and weakness of the centralized vaccination mode in rural areas? |
| 3. To make it well-run in rural areas, what do you suggest to optimize or improve the current vaccination policy?  (health human resource allocation, finance funding, financial incentives, village doctor’s involvement in vaccination service, regular sessions for routine vaccination service, vaccination days, home service, vaccination notice, vaccination appointment, etc ) |
| 4. How do you think about the economic incentive and encouragement of vaccination policy for vaccination professionals in township health center? |
| 5. How do you see the degree of village doctor’s participation in vaccination service? And how about professionals in your center? |
| 6. How do you think the attitudes of higher health authorities towards vaccination service? |
| 7. Meanwhile, you are responsible for the national essential public health services. How can you well manage the job of vaccination service? |

**Appendix S5 Measurement of independent variables**

Left-behind children (LBC) were defined as children living under the care of grandparents or other relatives whose parents had migrated elsewhere to work for at least 6 months at the time of the interview. Non-left-behind children (NLBC) were defined as children living with at least one of their parents at the time of the interview. For LBC, the primary guardian was any non-parental adult relative and for NLBC, one of the child’s parents.

Household registration status was determined based on whether the child was registered in the national household registration system at the time of the interview. Family income was divided into tertile based on the annual per capita family income.

Status of pre-vaccination physical examination was determined based on whether the child received physical examination before vaccination, provided by the healthcare worker. Status of post-vaccination advice was determined based on whether the child received the doctor’s advice after vaccination.

Primary guardian’s knowledge on measles vaccination, perception of susceptibility to measles, perception of severity in measles, perception of benefit from measles vaccination, perception of barriers to vaccination, perception of cues to action, perception of self-efficacy, practice towards measles vaccination and satisfaction with vaccination service were divided into two groups based on the median score of each domain. Five-point Likert-scale approaches were adopted to measure the primary guardians’ attitudes and health beliefs toward measles vaccination and their satisfaction with the vaccination service.

Based on whether the travel time taken to reach a township hospital was more than or equal to 30 minutes, villages were classified into two groups. In addition, villages were grouped into two teams based on whether village doctors were involved in routine measles vaccination service.

Township hospitals were classified into two groups based on whether a full-time vaccination workgroup was established, whether the allocation standard for vaccination professionals was met, whether vaccination service was provided on local market days, whether vaccination notice sheet was monthly offered, whether formal vaccination appointment was made with child’s guardian, or whether regular sessions for routine vaccination were uniformly distributed over a month.

**Appendix S6 Parameter estimation for null models at different levels**

| **Type of model** | **Parameter** | **MCV1** | | | |  | **MCV2** | | | |
| --- | --- | --- | --- | --- | --- | --- | --- | --- | --- | --- |
|  |  | **Estimate** | **Standard**  **error** | **χ^2^** | ***P* value** |  | **Estimate** | **Standard**  **error** | **χ^2^** | ***P* value** |
| One-level model | Fixed part |  |  |  |  |  |  |  |  |  |
|  | Constant | -0.411 | 0.059 | 48.275 | < 0.001 |  | -1.386 | 0.089 | 242.519 | < 0.001 |
| Two-level model | Fixed part |  |  |  |  |  |  |  |  |  |
|  | Constant | -0.468 | 0.082 | 32.449 | < 0.001 |  | -1.633 | 0.110 | 220.387 | < 0.001 |
|  | Random part |  |  |  |  |  |  |  |  |  |
|  | Village level variance | 0.370 | 0.104 | 12.582 | < 0.001 |  | 0.611 | 0.184 | 11.022 | < 0.001 |
|  | Household level scale parameter | 1.000 | 0.000 | - | - |  | 1.000 | 0.000 | - | - |
| Three-level model | Fixed part |  |  |  |  |  |  |  |  |  |
|  | Constant | -0.474 | 0.143 | 10.922 | < 0.001 |  | -1.652 | 0.188 | 77.215 | < 0.001 |
|  | Random part |  |  |  |  |  |  |  |  |  |
|  | Township level variance | 0.322 | 0.131 | 6.069 | 0.013 |  | 0.533 | 0.225 | 5.612 | 0.017 |
|  | Village level variance | 0.080 | 0.073 | 1.202 | 0.272 |  | 0.136 | 0.127 | 1.147 | 0.284 |
|  | Household level scale parameter | 1.000 | 0.000 | - | - |  | 1.000 | 0.000 | - | - |
